# Analysis of the relationship between color and natural pigments of tobacco leaves during curing

**DOI:** 10.1038/s41598-023-50801-1

**Published:** 2024-01-02

**Authors:** Yang Meng, Yuanhui Wang, Weimin Guo, Ke Lei, Zuxiao Chen, Hang Xu, Aiguo Wang, Qiang Xu, Jianjun Liu, Qiang Zeng

**Affiliations:** 1 College of Food Science and Engineering, Henan University of Technology, No. 100 Lianhua Street, Zhengzhou, 450001 China; 2 Zhengzhou Tobacco Research Institute of CNTC, Zhengzhou, 450001 China; 3 Henan Provincial Tobacco Company, Zhengzhou, 450001 China; 4 Henan Province Wheat-Flour Staple Food Engineering Technology Research Centre, Henan University of Technology, Zhengzhou, 450001 China; 5 Nanping Branch of Fujian Provincial Tobacco Company, Nanping, 353000 China

**Keywords:** Biochemistry, Plant sciences

## Abstract

Color is one of the most important indicators for the flue-cured tobacco quality. The color change of tobacco has a great relationship with the natural pigments in the tobacco. The relationship between color characteristics and the content of natural pigments in tobacco leaves during curing was investigated. The middle part of variety K326 tobacco was taken at each key time point during the curing process to determine the changes of color characteristics, moisture, pigment and polyphenol content. The results showed that moisture content of wet basis of tobacco gradually decreased from 72 to 18% during the curing process, the b* value increased and then decreased, and the a* value increased significantly. The lutein and *β*-carotene content decreased to 63.83 μg/g and 28.3 μg/g, respectively. The total polyphenols content increased to 50.19 mg/g. Meanwhile, the a* value was significantly and positively correlated with polyphenols content and negatively correlated with pigments content. Cluster analysis showed that the samples were divided into three categories: samples with the curing time of 0 h, 24–72 h, and 84–132 h. These results demonstrated that the color change of tobacco during curing process can be divided into three stages from the perspective of chemical composition, which are strongly related to the degradation of pigments and the transformation of polyphenols.

## Introduction

In the growth, development and maturity stage of plants, the change of color has been widely concerned by people^1^. The change of plant appearance color not only involves the regulation of proteins and other molecules, but also involves a series of complex biological processes such as material metabolism. The color change of tobacco curing has always been the focus of research in the tobacco industry^2,3^. Color change is an important index of flue-cured tobacco quality^4,5^. Moreover, the color of tobacco leaves is related to the sensory quality, and it is also an important factor for tobacco farmers to pick flue-cured tobacco leaves^6^.

Tobacco curing process from the appearance of the color change and moisture loss, reasonable tobacco curing is a process of tobacco moisture loss and yellowing coordination. The main physiological and biochemical processes include the decomposition and transformation of carbohydrates, proteins and pigments, the formation of aroma-inducing substances, and the change of polyphenol oxidase activity^7,8^. The color of tobacco leaves is determined by a variety of coloring substances, such as pigments. According to the chemical structure, the pigments contained in tobacco can be divided into the following four categories: pyrrole pigments represented by chlorophyll, polyene pigments represented by carotenoids, phenolic pigments represented by flavonoids, and ketone pigments represented by betaine. The color of tobacco leaves is the comprehensive expression of pigments in fresh leaves and coloring substances formed by browning reaction during curing. The plant pigments in fresh tobacco leaves are mainly composed of chlorophyll, *β*-carotene, neoxanthin and violaxanthin^9,10^.

Pigments are one of the most important influencing factors in the formation of color during tobacco curing^11^. After curing, the pigments inside the tobacco leaves are decomposed and transformed, which will affect the color of the tobacco leaves^12^. The yellowing of tobacco is a reflection of organic substance transformation, which is an enzymatic process. Moreover, the appearance of tobacco color from green to yellow indicates that the enzymatic process has been completed. Meanwhile, with the decrease of moisture content in the tobacco during curing, the enzyme activity is weakened or terminated, which will lead to the decrease of the change rate of chemical substances, and the yellow color of the tobacco is fixed^13^. Throughout the curing process, chlorophyll degrades completely with time, while the proportion of carotenoid in the total pigment increases gradually with curing time and develops to a dominant position^10^. The appearance of tobacco leaves at the end of curing is yellow. In addition, carotenoid is an important source of tobacco flavor and has a significant effect on tobacco quality. Thus, carotenoid was chosen to study the relationship between tobacco color and pigments. Furthermore, the molecular mechanism of tobacco leaf pigment metabolism was studied using isobaric tags for relative and absolute quantification (iTRAQ) proteomics approach^2^.

Phenolic pigments are polyphenol derivatives, also known as plant polyphenols^14,15^. Polyphenols have a great influence on the color change of tobacco leaves. Polyphenol oxidase (PPO) oxidizes polyphenols to quinone, which undergoes a series of reactions to produce brownish-yellow substances to realize the curing process of tobacco leaves from green to yellow. Therefore, it is necessary to effectively control the enzymatic oxidation of polyphenols during curing to improve the appearance quality of tobacco leaves. Meanwhile, the temperature and time of curing also influence the decomposition and transformation of pigments, which affects the color of tobacco leaves^16,17^. The previous study reported that the retention rates of Maillard reaction compounds and carotenoid degradation products decreased by 14.5% and 9.4% with raising the temperature (10 °C)^18^. Therefore, the parameters of curing need to be controlled to obtain the desired color and quality of tobacco.

In this study, the middle part of tobacco leaves variety K326 produced in Nanping, Fujian Province, China, were used as test materials. The aim is to characterize the relationship between color characteristics and pigments content in tobacco leaves during curing through analysis of variance (ANOVA), correlation analysis, principal component analysis (PCA) and cluster analysis, which can provide a basis for further optimization of tobacco curing process.

## Materials and methods

### Plant materials

Variety K326 was planted in Nanping City, Fujian Province, China. Before sampling, tobacco leaves with principally the same growth, relatively consistent leaf color and leaf size, and principally the same field quality were selected as test materials. The tobacco leaves from the middle part were taken and cured generally in the tobacco baking room with a three-stage curing process. The process was divided into three stages: yellowing stage, color fixing stage and dry tendon stage. The key temperature point parameters and tobacco change targets during the curing process are shown in Table 1^19^.

**Table 1:** The key temperature point parameters in three-stage curing process.

Curing stage	Dry bulb temperature/°C	Wet bulb temperature/°C	Heating up time/h	Temperature stabilization time/h	Tobacco change target	
					Color change	Drying change
Yellowing stage	38	36.5	5	15	Leaves 70 to 80% yellow	Leaves become soft
	40	38	4	12	Leaves 90% yellow	Leaves become soft
	42	38	4	15	Whole leaves yellow	Main vein becomes soft
Setting the color stage	45	38.5	6	18	Main vein turns white	Leaves blade hooked tip and rolled edge
	54	39	9	22	Main vein turns white	Total leaf drying
Dry tendon stage	60	40	6	4	Main vein turned purple	Partial drying of main vein
	68	41	8	4	Main vein turned purple	Dryness of main vein

During the curing process, tobacco samples were collected from the upper, middle and lower layers of the tobacco baking room once at 24 h, and after collecting twice, once at 12 h until the leaves were dry. A total of ten groups of tobacco samples were taken, forty pieces in each group. Ten pieces were used for color determination, and ten pieces were used for moisture determination. After removing the main vein, twenty pieces were freeze-dried using a freeze-dryer (FreeZone2.5Plus, Labconco, USA) before the determination of pigments and polyphenols.

### Analysis of samples

#### Determination of the color

During the curing process, a portable spectrophotometer (Ci64, X-rite, USA) was used to measure the lightness value (L*), greenness/redness value (a*), blueness/yellowness value (b*), color ratio (H*), hue (H°), saturation (C^*^) and color difference (ΔE) of ten pieces of tobacco leaves selected from each time point. According to the previously reported method, each tobacco leaf was determined at six measurement points^20^.

#### Determination of moisture content of wet basis

Tobacco leaves were placed in an oven and the moisture content of wet basis was determined using the heated-drying method. Ten pieces of tobacco leaves were dried at 105 ± 3 °C for at least 2 h, and the moisture content of wet basis of tobacco leaves was determined. The moisture content of wet basis is derived from the difference in mass of the tobacco before and after oven drying divided by the mass before oven drying^21^.

#### Determination of pigment content

A sample (2 g) was accurately weighed into the conical flask (50 mL), mixed with 25 mL 90% acetone, and sonicated for 20 min. Next, approximately 2 mL of the mixture was removed from the flask and filtered through a 0.45 µm organic membrane, with the filtrate collected in a high-performance liquid chromatography (HPLC) vial. The content of lutein and *β*-carotene in the filtrate were determined using HPLC^22^.

Determination conditions of HPLC: pigments were separated on a reversed-phase C_18_ column (Particle size 4 μm, 3.9 mm i.d. × 150 mm), with a mobile phase of (A) isopropanol and (B) acetonitrile (80%) using a gradient elution at a flow rate of 1.5 mL/min. The optimum elution gradient was as follows: 0–40 min, 100% B; 40–46 min, 100% A. The column temperature was 30 °C, and the injection volume was 10 μL. The detection wave length was 448 nm.

#### Determination of polyphenol content

A sample (0.1 g) was accurately weighed into the conical flask (50 mL), mixed with 20 mL 50% methanol, and sonicated for 20 min. Next, approximately 2 mL of the mixture was removed from the flask and filtered through a 0.45 µm hydrophilic membrane, with the filtrate collected in a HPLC vial. The content of chlorogenic acid, neochlorogenic acid, caffequinic acid, scopoletin, rutin and kaempferol glycosides in the filtrate was determined using HPLC^23,24^.

Determination conditions of HPLC: polyphenols were separated on a reversed-phase C_18_ column (Particle size 5 μm, 4.6 mm i.d. × 250 mm), with a mobile phase of (A) water–methanol-acetic acid (10:88:2, v/v/v) and (B) water–methanol-acetic acid (88:10:2, v/v/v) using a gradient elution at a flow rate of 1 mL/min. The optimum elution gradient was as follows: 0–16.5 min, 100% A, 16.5–30 min, 80% A + 20% B, 30–40 min, 20% A + 80% B. The column temperature was 30 °C, and the injection volume was 10 μL. The detection wave length was 340 nm.

### Data analysis

All experiments were carried out in triplicate according to independent replicate experiments, and the results were reported as mean value ± standard deviation. Significant difference analysis was executed by one-way ANOVA using SPSS Statistics 21.0 (SPSS Inc., Shanghai, China), Duncan’s test was used in post hoc analysis, and a probability value (*P* ≤ 0.05) indicated that the difference between the means was statistically significant. Origin 2021 software (OriginLab Corporation, USA) was used to operate PCA and draw correlation heatmap and hierarchical clustering heatmap.

### Ethics declarations

The authors declare that the collection of plant material (tobacco leaves) and the experimental studies complied with the relevant institutional, national and international guidelines and legislation. The authors confirm that all methods were carried out in accordance with relevant guidelines in the method section. The authors comply with the IUCN Policy Statement on Research Involving Species at Risk of Extinction and the Convention on the Trade in Endangered Species of Wild Fauna and Flora.

The authors ensure that the collection of tobacco leaves samples had been licensed by the local tobacco production regulatory agency.

## Results and discussion

### Significance analysis

#### Color change of tobacco leaves

With the progress of curing in tobacco leaves, the indexes of color were significantly different before and after 24 h in Table 2. In the whole process of curing, the difference among the upper, middle and lower layers of color was not significant in tobacco baking room. With the increase of curing time, the L* value and the b* value of the tobacco leaves first increased and then decreased. The former achieved the maximum value (71.8) at 72 h and the latter (59.0) at 24 h. While the a* value of tobacco leaves progressively increased. The C* value and the H° value also increased and then decreased, both of which reached the maximum at 24 h. The H* was rising, while the ΔE value first rose and then stabilized after 24 h. The results are consistent with the drying process results of Radix Paeoniae Alba (RPA) slice^25^. The L* value decreased as the moisture content of wet basis decreased, while the a* value and the b* value increased with the decrease in the moisture content of wet basis. On the whole, the color of tobacco leaves changed significantly from green to yellow in the early stage (from 0 to 72 h), and the yellowness of tobacco leaves deepened in the later stage (from 72 to 132 h).

**Table 2:** The changes of color in tobacco leaves under different curing time.

Indicators	Location	Curing time (h)									
		0	24	48	60	72	84	96	108	120	132
L*	Upper layer	54.84 ± 4.22^k^	67.89 ± 2.21^bcde^	70.39 ± 1.25^abc^	70.52 ± 0.78^ab^	71.79 ± 1.26^a^	68.28 ± 1.83^bcd^	67.25 ± 0.81^cdefg^	62.85 ± 0.42^hij^	65.22 ± 0.26^defgh^	61.68 ± 0.14^ij^
	Middle layer	56.06 ± 2.94^k^	72.08 ± 0.87^a^	70.26 ± 1.55^abc^	70.10 ± 0.79^abc^	71.9 ± 0.43^a^	68.34 ± 1.10^bcd^	65.27 ± 1.08^defgh^	63.95 ± 1.38^hij^	64.56 ± 1.80^fghi^	61.02 ± 1.25^j^
	Lower layer	56.64 ± 3.97^k^	70.12 ± 3.20^abc^	70.45 ± 0.42^abc^	69.16 ± 0.18^abc^	68.21 ± 0.78^bcd^	67.55 ± 1.03^bcdef^	65.02 ± 0.16^efgh^	64.41 ± 0.49^ghi^	64.26 ± 0.26^ghi^	61.78 ± 1.24^ij^
a*	Upper layer	− 9.86 ± 1.15^m^	− 0.60 ± 0.45^l^	4.83 ± 1.14^hij^	3.79 ± 0.30^j^	4.72 ± 0.79^hij^	4.77 ± 0.94^hij^	6.27 ± 0.63^defg^	6.82 ± 0.89^cdef^	6.80 ± 0.28^cdef^	8.35 ± 0.72^b^
	Middle layer	− 9.56 ± 1.05^m^	3.44 ± 0.69^j^	5.38 ± 0.56^eghi^	4.03 ± 0.73^ij^	4.02 ± 0.47^ij^	5.97 ± 0.65^efgh^	6.56 ± 1.31^defg^	7.43 ± 1.20^bcde^	8.04 ± 0.44^bc^	7.56 ± 0.31^bcd^
	Lower layer	− 9.95 ± 1.3^m^	1.93 ± 0.42^k^	5.29 ± 0.75^ghi^	4.68 ± 0.57^hij^	6.65 ± 0.89^cdefg^	6.29 ± 1.01^defg^	7.01 ± 0.57^bcde^	7.61 ± 0.24^bcd^	8.40 ± 0.27^b^	9.82 ± 0.29^a^
b*	upper layer	40.93 ± 3.32^m^	58.11 ± 1.43^a^	52.81 ± 2.16^bc^	52.83 ± 1.53^bc^	51.43 ± 3.53^cdefg^	49.16 ± 2.23^defghi^	48.29 ± 0.56^fghij^	47.10 ± 1.29^hi^j	50.25 ± 1.06^cdefgh^	45.83 ± 0.23^ijk^
	Middle layer	42.38 ± 2.06^lm^	57.84 ± 0.78^a^	55.87 ± 3.08^ab^	50.96 ± 1.01^cdefg^	52.20 ± 0.72^cde^	49.92 ± 0.95^cdefgh^	46.95 ± 2.55^hij^	48.05 ± 2.30^ghij^	49.53 ± 0.34^cdefgh^	45.17 ± 0.83^jkl^
	Lower layer	43.18 ± 2.49^klm^	59.07 ± 2.47^a^	52.57 ± 2.77^cd^	48.58 ± 1.10^efghij^	51.71 ± 2.55^cdef^	50.58 ± 0.12^cdefgh^	47.83 ± 0.64^ghij^	49.49 ± 1.98^cdefgh^	49.54 ± 0.84^cdefgh^	48.99 ± 0.35^defghi^
Color ratio	Upper layer	− 0.24 ± 0.05^l^	− 0.01 ± 0.01^k^	0.09 ± 0.02^efghi^	0.07 ± 0.00^hij^	0.09 ± 0.02^efghi^	0.10 ± 0.02^defghi^	0.13 ± 0.01^bcdefg^	0.14 ± 0.02^abcde^	0.14 ± 0.01^bcdef^	0.18 ± 0.02^ab^
	Middle layer	− 0.23 ± 0.04^l^	0.06 ± 0.01^ij^	0.10 ± 0.00^efghi^	0.08 ± 0.01^fghij^	0.08 ± 0.01^ghij^	0.12 ± 0.01^cdefgh^	0.14 ± 0.02^bcde^	0.15 ± 0.02^abcd^	0.16 ± 0.01^abc^	0.17 ± 0.01^abc^
	Lower layer	− 0.23 ± 0.04^l^	0.03 ± 0.01^jk^	0.10 ± 0.010^defghi^	0.10 ± 0.01^efghi^	0.13 ± 0.01^bcdefgh^	0.12 ± 0.02^cdefgh^	0.15 ± 0.01^abcde^	0.15 ± 0.00^abcd^	0.17 ± 0.00^abc^	0.20 ± 0.01^a^
Hue	Upper layer	− 1.33 ± 0.05^j^	− 1.56 ± 0.01^k^	1.48 ± 0.02^bcde^	1.50 ± 0.00^abc^	1.48 ± 0.02^bcde^	1.47 ± 0.02^bcde^	1.44 ± 0.01^defgh^	1.43 ± 0.02^efghi^	1.44 ± 0.01^efgh^	1.39 ± 0.01^hi^
	Middle layer	− 1.35 ± 0.03^j^	1.51 ± 0.01^ab^	1.47 ± 0.00^bcde^	1.49 ± 0.01^abcd^	1.49 ± 0.01^abcd^	1.45 ± 0.01^cdefg^	1.43 ± 0.02^efgh^	1.42 ± 0.02^fghi^	1.41 ± 0.01^ghi^	1.40 ± 0.01^ghi^
	Lower layer	− 1.34 ± 0.04^j^	1.54 ± 0.01^a^	1.47 ± 0.01^bcdef^	1.47 ± 0.01^bcde^	1.44 ± 0.01^defgh^	1.45 ± 0.02^cdefg^	1.43 ± 0.01^efghi^	1.42 ± 0.00^fghi^	1.40 ± 0.00^ghi^	1.37 ± 0.01^i^
Saturation	Upper layer	42.13 ± 2.95^j^	58.12 ± 1.43^ab^	53.04 ± 2.25^bcde^	52.97 ± 1.54^bcde^	51.65 ± 3.53^defg^	49.40 ± 2.24^efgh^	48.70 ± 0.54^efghi^	47.60 ± 1.38^efghij^	50.71 ± 1.060^defg^	46.58 ± 0.30^fghij^
	Middle layer	43.46 ± 1.77^ij^	57.94 ± 0.76^abc^	56.13 ± 3.12^abcd^	51.13 ± 1.06^defg^	52.36 ± 0.75^bcdef^	50.28 ± 1.01^defg^	47.42 ± 2.70^efghij^	48.63 ± 2.44^efghi^	50.18 ± 0.41^efgh^	45.80 ± 0.81^ghij^
	Lower layer	44.34 ± 2.18^hij^	59.10 ± 2.46^a^	52.84 ± 2.82^bcde^	48.81 ± 1.15^efghi^	52.14 ± 2.63^cdef^	50.98 ± 0.11^defg^	48.34 ± 0.70^efghi^	50.07 ± 1.98^efgh^	50.25 ± 0.87^defgh^	49.96 ± 0.36^efgh^
ΔE	Upper layer	0.00^b^	23.6 ± 7.49^a^	24.54 ± 2.86^a^	24.03 ± 5.04^a^	24.94 ± 5.30^a^	21.93 ± 5.88^a^	21.73 ± 4.11^a^	19.82 ± 4.38^a^	21.88 ± 4.49^a^	20.42 ± 3.26^a^
	Middle layer	0.00^b^	25.81 ± 3.67^a^	24.68 ± 1.48^a^	21.36 ± 2.79^a^	23.09 ± 3.96^a^	21.21 ± 2.10^a^	19.34 ± 1.64^a^	19.81 ± 3.25^a^	20.85 ± 2.61^a^	18.37 ± 2.90^a^
	Lower layer	0.00^b^	24.41 ± 4.60^a^	22.85 ± 5.39^a^	20.14 ± 4.50^a^	22.22 ± 5.47^a^	21.08 ± 3.58^a^	19.71 ± 4.01^a^	20.52 ± 3.64^a^	21.06 ± 2.83^a^	21.64 ± 3.02^a^

#### Moisture content of wet basis change of tobacco leaves

**Figure 1:**
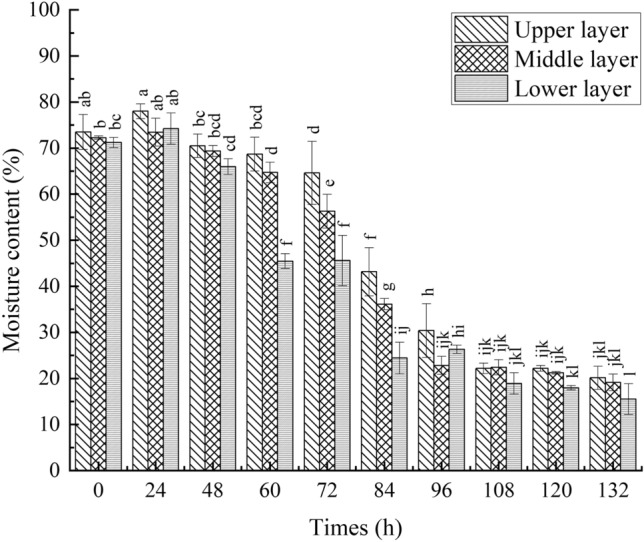
The change of the moisture content of wet basis in tobacco leaves under different curing time. Different letters indicated significant differences at different times (*P* ≤ 0.05).

The moisture content of wet basis of the upper, middle and lower layers of tobacco leaves in tobacco baking room was significantly different in Fig. 1. From the beginning to the end of curing, the moisture content of wet basis of the tobacco leaves decreased to 15–20%. Before the curing time (72 h), the moisture content of wet basis changed little. The moisture content of wet basis decreased substantially from the curing time 72 h to 108 h. After that, the moisture content of wet basis tended to be stable. These results indicated that the water loss of tobacco leaves during the curing process was slow in the early stage (from 0 to 72 h), accelerated in the middle stage (from 72 to 96 h), and stabilized in the later stage (from 96 to 132 h). In the study of the moisture distribution and state of stemless tobacco leaves during curing, the moisture content of wet basis was about 10%. The plant material was Variety Yunyan 87, and it was planted in Luoyang City, Henan Province^26^. The equilibrium moisture content was lower in Luoyang tobacco and higher in Fujian^27^. The experimental results of Yunyan 87 were different from the results of this experiment, it might be caused by the different humidity in the two places^28^.

#### The change of lutein and *β*-carotene content in tobacco leaves

**Figure 2:**
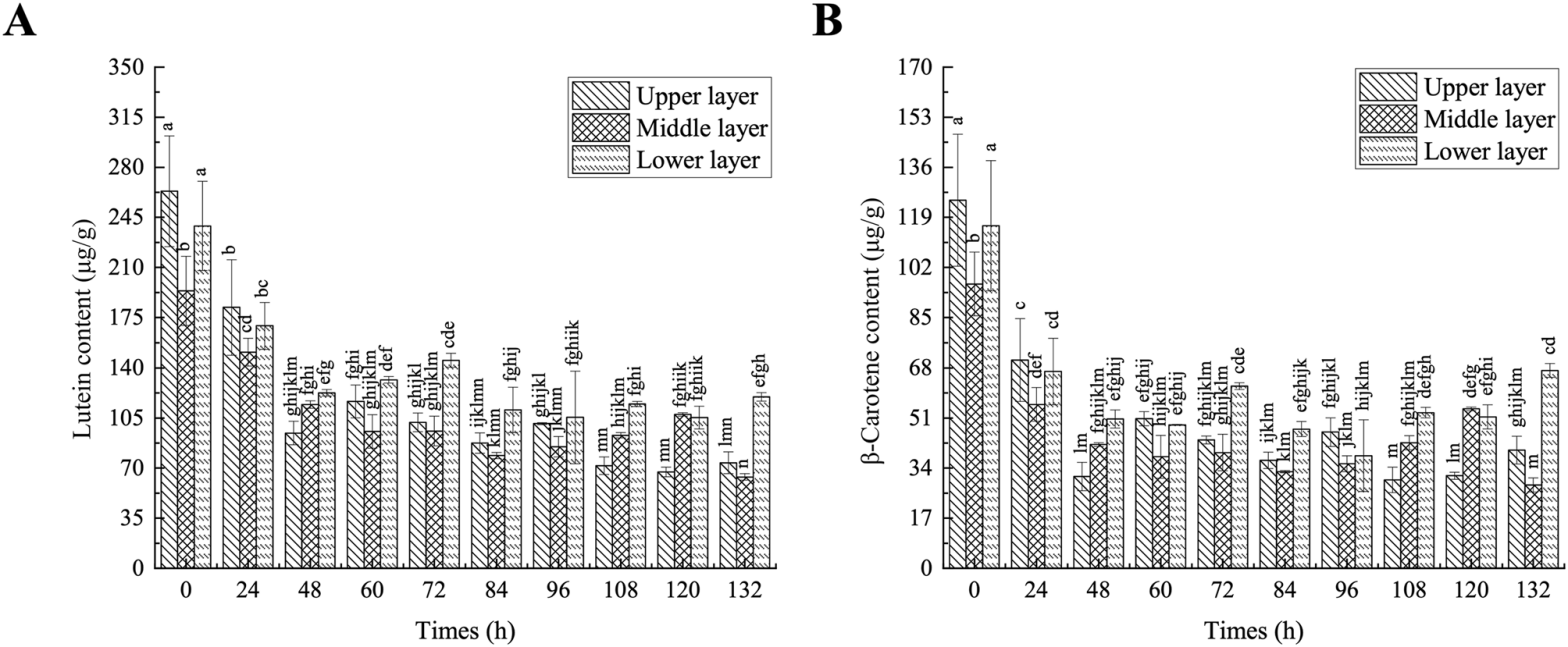
The changes of plastid pigments in tobacco leaves under different curing time. Different letters indicated significant differences at different times (*P* ≤ 0.05). (**A**) The change of the lutein content in tobacco leaves under different curing time. (**B**) The change of the *β*-carotene content in tobacco leaves under different curing time.

From the beginning to the end of tobacco curing, the difference between the upper, middle and lower layers of pigments content was significant in tobacco baking room in Fig. 2, and the lutein and *β*-carotene content was significantly higher in the upper and lower layers than in the middle layer. This may be caused by uneven heating of tobacco leaves at different positions in tobacco baking room^29^. The lutein content of the tobacco leaves decreased continuously with the increase of curing time. The content of *β*-carotene and lutein decreased rapidly until 48 h, while the content decreased slowly after that. The results demonstrated that the trend in *β*-carotene content followed that in lutein content. During the curing process, the substances of tobacco leaves were oxidized and decomposed by the action of lipoxygenase, and intermediate products such as violaxanthin, geraniol and violetone were formed^11^. The decrease in the content of both may be related to this reason.

#### Polyphenols changes of tobacco leaves

During the whole process of tobacco curing, the polyphenols content was not significantly different in the upper, middle and lower layers of the tobacco baking room in Table 3. With the increase of curing time, the content of chlorogenic acid, neochlorogenic acid and caffequinic acid in tobacco leaves increased continuously. The content of rutin and kaempferol glycoside in tobacco leaves first increased and then decreased, reaching the maximum value between 96 and 108 h. The content of scopoletin in tobacco leaves showed a trend of first increasing, decreasing and then increasing, reaching the great value with 48 h and 120 h, respectively. In addition, the total polyphenols content showed a trend of increasing. The changes of polyphenolic compounds were very intense during the curing period of tobacco leaves, and the total polyphenolic compounds increased significantly due to the pyrolysis and enzymatic decomposition of phenol glycosides^30^. Polyphenols can be pyrolyzed into dibasic phenolic and furfural derivatives. Moreover, polyphenols are easily oxidized to produce light red to dark brown quinones and their polymers under the action of peroxidase and polyphenol oxidase, resulting in the color change of tobacco leaves from yellow to orange or different degrees of brownish-brown. The polyphenolic substances and their oxidation products can also occur with amino acids, sugars and minerals enzymatic browning reaction, forming many complex pigments of different molecular weight and color, which also have a vital impact on the formation of tobacco color^31^.

**Table 3:** The changes of polyphenols in tobacco leaves under different curing time.

Indicators (μg/g)	Location	Curing time (h)									
		0	24	48	60	72	84	96	108	120	132
Neochlorogenic acid content	Upper layer	1.59 ± 0.23^kl^	1.81 ± 0.22^ijk^	1.73 ± 0.11^igkl^	2.32 ± 0.18^fg^	2.01 ± 0.22^hi^	2.47 ± 0.04^efg^	2.85 ± 0.47^bcd^	2.27 ± 0.21^gh^	2.74 ± 0.21^cde^	2.72 ± 0.07^cde^
	Middle layer	1.20 ± 0.06^m^	1.88 ± 0.02^ijk^	1.64 ± 0.11^jkl^	1.94 ± 0.13^ij^	1.86 ± 0.02^ijk^	2.53 ± 0.12^efg^	2.60 ± 0.16^cdef^	2.67 ± 0.19^cde^	3.12 ± 0.01^b^	2.54 ± 0.05^defg^
	Lower layer	1.48 ± 0.04^l^	1.78 ± 0.02^ijk^	2.00 ± 0.21^hi^	2.24 ± 0.00^gh^	2.61 ± 0.04^cde^f	2.69 ± 0.13^cde^	2.73 ± 0.25^cde^	2.63 ± 0.27^cdef^	2.90 ± 0.06^bc^	3.43 ± 0.11^a^
Caffequinic acid content	Upper layer	2.07 ± 0.13^m^	2.56 ± 0.33^hijkl^	2.22 ± 0.02^lm^	2.86 ± 0.07^efgh^	2.65 ± 0.10^ghij^	3.07 ± 0.05^def^	3.32 ± 0.66^cd^	2.76 ± 0.20^fghi^	3.19 ± 0.12^cde^	3.17 ± 0.09^cde^
	Middle layer	1.58 ± 0.03^n^	2.30 ± 0.02^jklm^	2.41 ± 0.10^ijklm^	2.43 ± 0.25^ijklm^	2.47 ± 0.05^ijkl^	3.10 ± 0.16^def^	2.97 ± 0.07^defg^	3.03 ± 0.24^def^	3.87 ± 0.01^b^	3.01 ± 0.17^defg^
	Lower layer	2.27 ± 0.20^klm^	2.43 ± 0.04^ijklm^	2.77 ± 0.30^fghi^	2.64 ± 0.03^ghijk^	3.21 ± 0.02^cde^	3.32 ± 0.22^cd^	3.18 ± 0.21^cde^	3.17 ± 0.31^cde^	3.53 ± 0.16^c^	4.27 ± 0.13^a^
Chlorogenic acid content	Upper layer	11.17 ± 0.75^k^	16.15 ± 0.48^hij^	16.18 ± 1.01^hij^	15.51 ± 0.81^j^	16.66 ± 0.98^ghij^	18.74 ± 0.03^ef^	19.31 ± 1.03^de^	21.04 ± 1.41^c^	22.71 ± 0.41^b^	19.59 ± 0.29^cde^
	Middle layer	9.68 ± 0.45^l^	15.57 ± 0.61^j^	16.28 ± 0.54^hij^	15.67 ± 0.21^ij^	17.21 ± 0.29^ghi^	20.47 ± 0.98^cd^	19.43 ± 0.57^cde^	20.94 ± 0.50^c^	23.38 ± 0.02^ab^	20.17 ± 1.05^cde^
	Lower layer	11.47 ± 0.17^k^	16.77 ± 1.56^ghij^	16.62 ± 1.12^ghij^	17.41 ± 0.09^fgh^	17.85 ± 0.47^fg^	20.77 ± 1.35^cd^	20.56 ± 0.63^cd^	20.11 ± 0.13^cde^	20.01 ± 2.05^cde^	24.30 ± 0.55^a^
Scopoletin content	Upper layer	0.19 ± 0.01^j^	0.30 ± 0.01^bcd^	0.29 ± 0.03^cdef^	0.23 ± 0.01^hi^	0.22 ± 0.01^i^	0.29 ± 0.00^cde^	0.26 ± 0.00^efg^	0.24 ± 0.02^ghi^	0.27 ± 0.00^def^	0.29 ± 0.00^cd^
	Middle layer	0.17 ± 0.01^j^	0.26 ± 0.01^fgh^	0.32 ± 0.02^ab^	0.24 ± 0.01^ghi^	0.29 ± 0.01^cdef^	0.31 ± 0.00^bc^	0.28 ± 0.00^cdef^	0.28 ± 0.00^cdef^	0.35 ± 0.00^a^	0.30 ± 0.01^bcd^
	Lower layer	0.22 ± 0.01^i^	0.26 ± 0.00^efg^	0.28 ± 0.04^cdef^	0.24 ± 0.03^ghi^	0.27 ± 0.01^def^	0.29 ± 0.02^cd^	0.29 ± 0.01^bcd^	0.30 ± 0.03^bcd^	0.29 ± 0.03^bcd^	0.29 ± 0.00^cd^
Rutin content	Upper layer	16.22 ± 3.29 lm	18.76 ± 0.19^hijk^	19.71 ± 1.24^efghij^	16.68 ± 0.29^klm^	18.98 ± 0.67^hij^	21.27 ± 1.18^cdefg^	20.28 ± 1.34^defghi^	24.82 ± 1.39^ab^	24.68 ± 0.25^ab^	20.83 ± 0.17^defgh^
	Middle layer	15.63 ± 1.24^m^	18.17 ± 0.97^ijkl^	19.30 ± 0.56^ghij^	19.00 ± 1.28^hij^	21.57 ± 0.09^cdef^	21.97 ± 0.14^cd^	21.54 ± 0.09^cdef^	22.26 ± 1.89^cd^	23.18 ± 0.02^bc^	22.12 ± 0.79^cd^
	Lower layer	16.60 ± 0.06 lm	17.59 ± 1.27^jklm^	17.57 ± 0.29^jklm^	19.42 ± 1.36^fghij^	19.65 ± 0.29^efghij^	20.2 ± 0.52^defghi^	25.53 ± 0.6^a^	20.79 ± 1.29^defgh^	21.68 ± 2.78^cde^	22.36 ± 0.39^cd^
Kaempferol glycoside content	Upper layer	1.42 ± 0.19^hi^	1.59 ± 0.00^efgh^	1.74 ± 0.15^def^	1.49 ± 0.06^gh^	1.76 ± 0.04^cde^	2.00 ± 0.12^b^	1.90 ± 0.02^bcd^	2.42 ± 0.22^a^	2.35 ± 0.02^a^	1.93 ± 0.03^bc^
	Middle layer	1.28 ± 0.05^i^	1.50 ± 0.08^gh^	1.57 ± 0.03^fgh^	1.61 ± 0.03^efg^	1.89 ± 0.02^bcd^	1.94 ± 0.08^bc^	2.08 ± 0.11^b^	2.09 ± 0.18^b^	2.26 ± 0.00^a^	2.05 ± 0.00^b^
	Lower layer	1.42 ± 0.00^hi^	1.48 ± 0.09^gh^	1.49 ± 0.02^gh^	1.91 ± 0.13^bcd^	1.92 ± 0.00^bcd^	1.98 ± 0.02^b^	2.29 ± 0.05^a^	1.97 ± 0.12^b^	2.04 ± 0.23^b^	1.94 ± 0.04^bc^
Total polyphenols content	Upper layer	32.67 ± 3.89^h^	41.17 ± 0.83^fg^	41.87 ± 2.29^fg^	39.1 ± 0.79^g^	42.29 ± 1.36^efg^	47.83 ± 1.18^cd^	47.92 ± 0.78^cd^	53.56 ± 3.45^ab^	55.94 ± 1.02^a^	48.52 ± 0.6^cd^
	Middle layer	29.54 ± 1.84^i^	39.68 ± 1.64^g^	41.51 ± 1.08^fg^	40.89 ± 0.70^fg^	45.30 ± 0.46^de^	50.32 ± 1.47^bc^	48.91 ± 0.14^c^	51.27 ± 2.15^bc^	56.16 ± 0.05^a^	50.19 ± 2.06^c^
	Lower layer	33.45 ± 0.36^h^	40.30 ± 2.90^g^	40.73 ± 1.4^fg^	43.85 ± 1.58^ef^	45.51 ± 0.75^de^	49.24 ± 1.22^c^	54.57 ± 0.83^a^	48.96 ± 0.98^c^	50.46 ± 5.31^bc^	56.6 ± 0.36^a^

### Correlation of color, moisture content of wet basis, pigments and polyphenols of tobacco leaves

**Figure 3:**
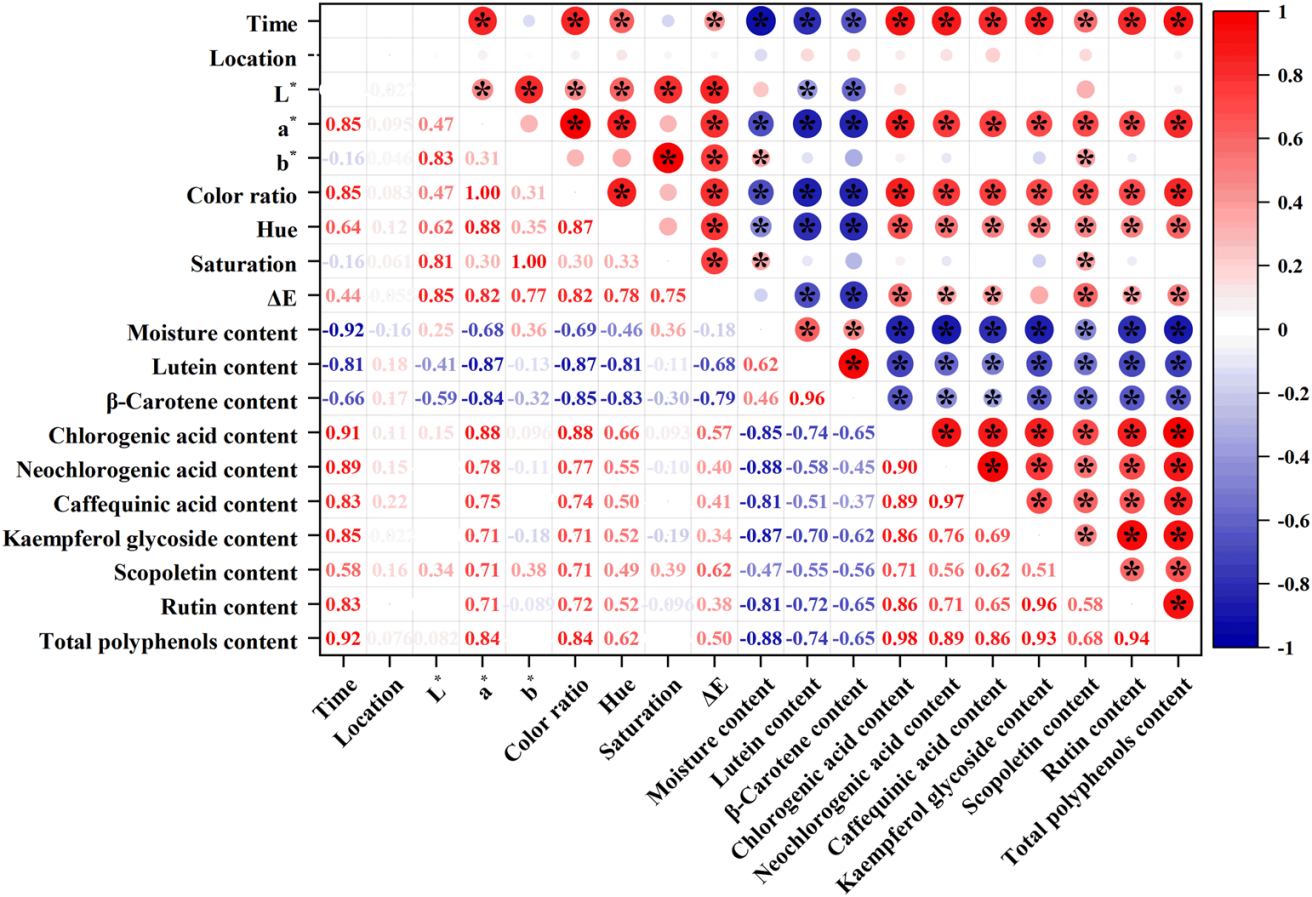
Heat map of the correlation between color, moisture content of wet basis, pigments and polyphenols content of tobacco leaves during curing (*P* ≤ 0.05).

As shown in Fig. 3, the color of tobacco leaves was significantly correlated with the moisture content of wet basis, pigments and polyphenols content during curing. The moisture content of wet basis of tobacco leaves was significantly and negatively correlated with polyphenols content, and it was significantly and positively correlated with the a* value. It could be due to the decrease in the moisture content of wet basis of tobacco leaves affects the activity of PPO and the content of malondialdehyde (MDA), which causes change in polyphenols content^28^. Meanwhile, the moisture content of wet basis was significantly and negatively correlated with the b* value. The a* value was significantly and positively correlated with polyphenols content and negatively correlated with pigments content. It may be due to the non-enzymatic browning (Maillard reaction). Due to the acceleration of Maillard reaction at higher temperatures, the interaction between sugars and amino acids is easy to form brown compounds, resulting in darker color^23^. The pigments content of tobacco leaves was significantly and negatively correlated with polyphenols content. Overall, the change in apparent color of tobacco leaves during curing was closely related to the content of intrinsic pigment classes.

### PCA

**Figure 4:**
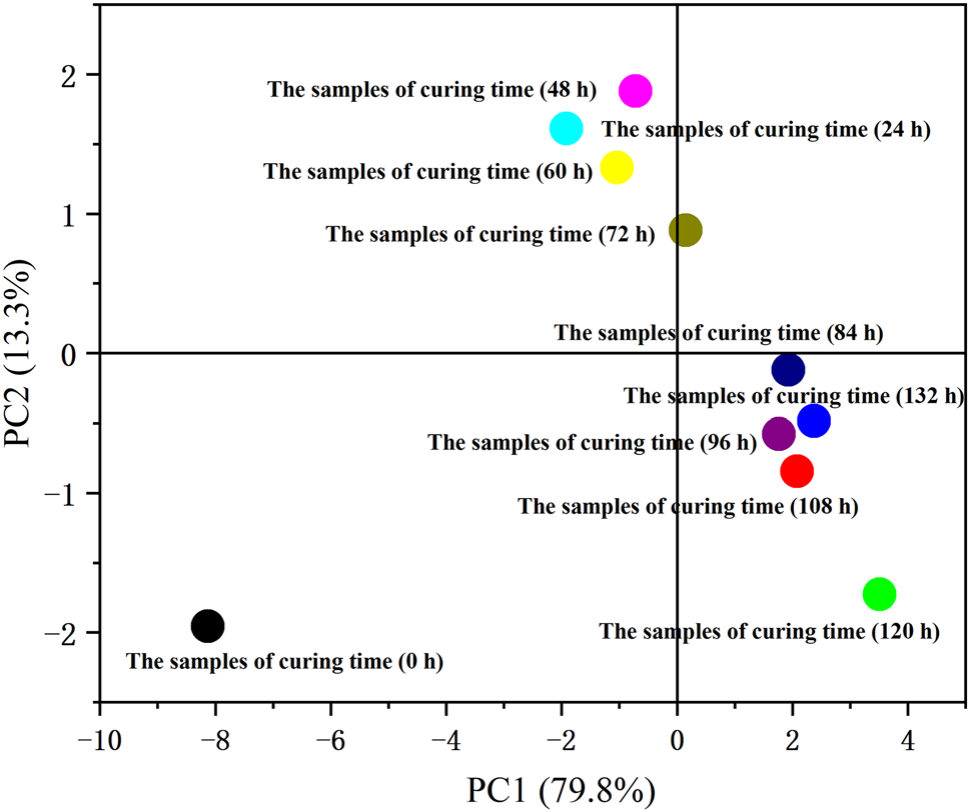
Plot of PCA scores derived from the samples of tobacco leaves during curing. In the figure, each curing time point represents the samples of various curing time.

Each index with a correlation coefficient greater than 0.50 was selected for PCA analysis with each curing time point. The main data information of the samples with different curing time points between each index was extracted by PCA to show the similarity between the samples. As shown in Fig. 4, the cumulative contribution of the first two principal components in the variance reached 93.1%, which indicated that these two principal components could represent most of the information on the curing characteristics of the samples. The PC1 was negatively correlated with the first four samples with curing time (0–60 h), while it was positively correlated with the other six samples (curing time of 72–132 h). In addition, the PC2 was positively correlated with the samples (curing time of 24–72 h), while it was negatively correlated with the other samples (curing time of 0 h and 84–132 h). The results showed that the curing characteristics of the samples with curing time (0–60 h) were statistically significantly different from those with curing time (72–132 h). This may be related to the yellowing stage of tobacco curing^32^. The yellowing stage starts from curing and generally takes from 24 to 72 h.

### Cluster analysis

Cluster analysis is one of the most important techniques for data mining and exploratory data analysis^33^. Each index with a correlation coefficient greater than 0.50 was selected for cluster with each curing time point.

**Figure 5:**
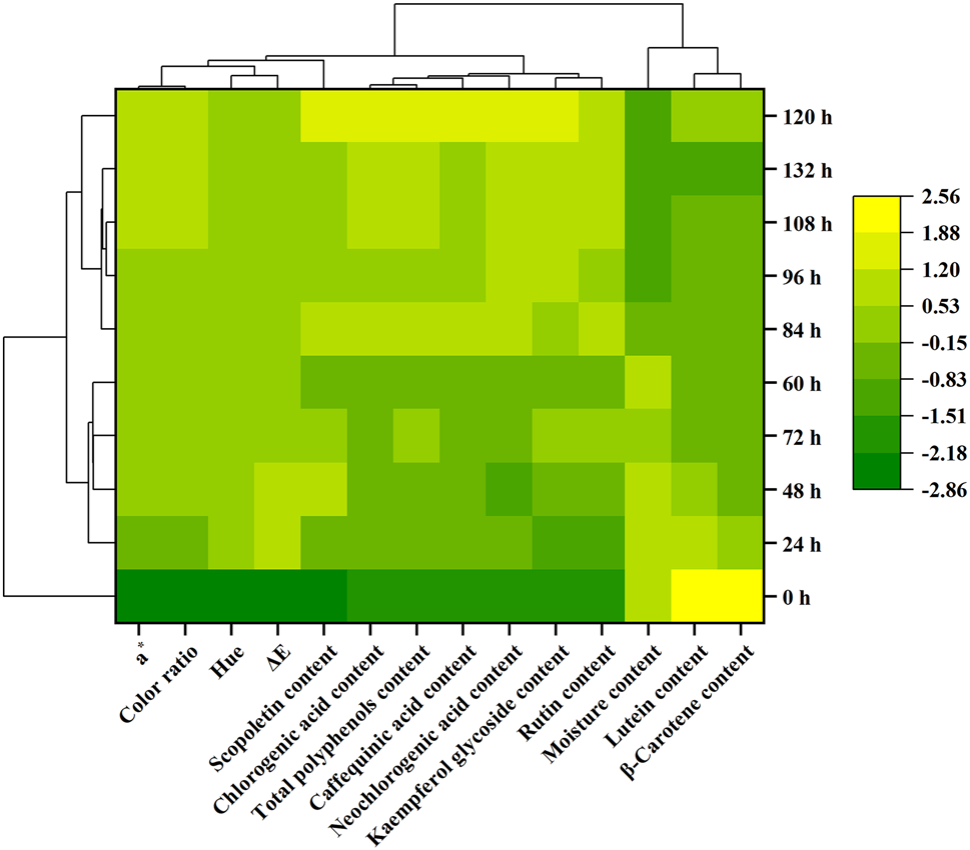
Cluster analysis of the samples of tobacco leaves during curing. In the figure, each curing time points represents the samples of various curing time.

In terms of each curing characteristic indicator, it could be divided into three categories (Fig. 5). The first category contained chromaticity and scopoletin content, the second category included the contents of chlorogenic acid, neochlorogenic acid, caffequinic acid, rutin, kaempferol glycosides and the total polyphenols, and the third category was composed of pigments content and moisture content of wet basis. Scopoletin content was not classified with other polyphenols contents, and it belonged to the coumarin class and had a different mechanism of action^28^. Moisture content of wet basis and pigments content were separated in one category. This was consistent with the analysis of significant differences. It showed that the results of moisture content of wet basis decrease and pigment degradation during curing were accurate and reliable.

From the samples of each curing time, they could be divided into three categories (Fig. 5). The first category contained samples with curing time (0 h), the second category included samples with curing time (24–72 h), and the third category was composed of samples with curing time (84–132 h). The results showed that tobacco curing could be divided into three stages. The color of fresh tobacco leaves changed from green to light yellow, and finally to brownish yellow. This corresponded to the yellowing stage, the color fixing stage and the dry tendon stage of tobacco curing^19^.

## Conclusions

The content of pigmented substances affected the color change of flue-cured tobacco to a certain extent. During the curing process, the lutein content and β-carotene content decreased to 63.83 μg/g and 28.3 μg/g, respectively. The total polyphenols content increased to 50.19 mg/g. Meanwhile, the a* value was significantly and positively correlated with the content of chlorogenic acid, neochlorogenic acid, caffequinic acid, scopoletin, rutin and kaempferol glycosides. The a* values of tobacco leaves were negatively correlated with lutein and *β*-carotene content. The PCA showed that the curing characteristics of the samples with curing time (0–60 h) were statistically significantly different from those with curing time (72–132 h). Cluster analysis showed that the samples were divided into three categories: samples with the curing time of 0 h, 24–72 h, and 84–132 h. The degradation of pigments and transformation of polyphenolic substances had a combined effect on the color of tobacco leaves during curing, causing a change in color to a deeper golden, deep yellow and brownish yellow. Properly controlling the degradation and conversion of pigmented substances will be helpful to make flue-cured tobacco reach the desired color.

## Data Availability

The data presented in this study are available on request from the corresponding author.
